# Dysbiosis of the microbiome in gastric carcinogenesis

**DOI:** 10.1038/s41598-017-16289-2

**Published:** 2017-11-21

**Authors:** Natalia Castaño-Rodríguez, Khean-Lee Goh, Kwong Ming Fock, Hazel M. Mitchell, Nadeem O. Kaakoush

**Affiliations:** 1 School of Biotechnology and Biomolecular Sciences, UNSW Sydney, Sydney, NSW 2052 Australia; 2 Department of Medicine, Faculty of Medicine, University of Malaya, Kuala Lumpur, Malaysia; 3 Division of Gastroenterology, Department of Medicine, Changi General Hospital, Singapore City, Singapore; 4 School of Medical Sciences, UNSW Sydney, Sydney, NSW 2052 Australia

## Abstract

The gastric microbiome has been proposed as an etiological factor in gastric carcinogenesis. We compared the gastric microbiota in subjects presenting with gastric cancer (GC, n = 12) and controls (functional dyspepsia (FD), n = 20) from a high GC risk population in Singapore and Malaysia. cDNA from 16S rRNA transcripts were amplified (515F-806R) and sequenced using Illumina MiSeq 2 × 250 bp chemistry. Increased richness and phylogenetic diversity but not Shannon’s diversity was found in GC as compared to controls. nMDS clustered GC and FD subjects separately, with PERMANOVA confirming a significant difference between the groups. *H. pylori* serological status had a significant impact on gastric microbiome α-diversity and composition. Several bacterial taxa were enriched in GC, including *Lactococcus*, *Veilonella*, and *Fusobacteriaceae* (*Fusobacterium* and *Leptotrichia*). Prediction of bacterial metabolic contribution indicated that serological status had a significant impact on metabolic function, while carbohydrate digestion and pathways were enriched in GC. Our findings highlight three mechanisms of interest in GC, including enrichment of pro-inflammatory oral bacterial species, increased abundance of lactic acid producing bacteria, and enrichment of short chain fatty acid production pathways.

## Introduction

Gastric cancer (GC) is the fifth most commonly diagnosed cancer and third leading cause of cancer-related deaths worldwide^1^. It accounted for an estimated 723,000 deaths (8.8% of total deaths from cancer) in 2012^1^. Almost two-thirds of GC cases occur in East Asia, Eastern Europe, and Central and South America, with Chinese ethnicity being identified as an independent risk factor for the development of GC in multiracial studies^2,3^. In countries such as Malaysia and Singapore, individuals with Chinese ethnicity have the highest age-standardized rate (ASR) (25.7 per 100,000 men) as compared with individuals of Malayan (6.6 per 100,000) and Indian (8.4 per 100,000) backgrounds^1^.

While *Helicobacter pylori* infection is the most important risk factor for GC, the etiology of GC is clearly multifactorial as evidenced by the fact that only 1–3% of *H. pylori*-infected patients develop GC, and that progression to GC in some subjects occurs even after eradication of the bacterium^4^. In addition to host and environmental factors, the gastric microbiome is now believed to contribute to the progression of disease, particularly when *H. pylori* disappears in patients who progress to the metaplastic and dysplastic stages. For example, Lertpiriyapong *et al*.^5^, using a transgenic *H. pylori* INS-GAS mouse model, reported that colonization with microbiota limited to three species of Altered Schaedler’s Flora, including ASF356 (*Clostridium* sp.), ASF361 (*Lactobacillus murinus*) and ASF519 (*Bacteroides* sp.), were sufficient to induce neoplasia. Further, a range of independent studies assessing gastric microbiota in relation to Correa’s Cascade have demonstrated that significant differences exist in the microbiota of those with gastritis, intestinal metaplasia (IM) and GC^6–10^, suggesting that dysbiosis in the stomach is dynamic and correlates with progression to gastric carcinogenesis.

In contrast, a study by Yang *et al*.^11^ which included subjects presenting with non-atrophic gastritis, AG and IM, from a high GC risk area (Túquerres) and a low GC risk area (Tumaco) in Colombia, failed to identify any correlation between gastric microbiota composition and histological diagnosis. However, the authors did identify that *Leptotrichia* spp. and *Veillonella* spp., were significantly more abundant in Túquerres while 16 OTUs, including a *Staphylococcus* spp., were significantly more abundant in Tumaco.

To our knowledge, no study to date has assessed the active microbiome (rRNA transcripts) in the context of gastric carcinogenesis. Here, we compared the active gastric microbiome of GC patients and functional dyspepsia (FD) controls using 16S rRNA transcript amplicon sequencing on an Illumina MiSeq platform, and identified a number of microbial changes and predicted functional changes associated with carcinogenesis.

## Methods

### Ethics approval and consent to participate

Ethics approval was obtained from the Human Ethics Committee (HREC) of the University of New South Wales (UNSW) (HREC 08115 and HREC 02144). All subjects recruited to the study signed the written informed consent. All experiments were performed in accordance with relevant guidelines and regulations.

### Subjects

This study comprised 36 predominantly ethnic Chinese subjects who underwent upper gastrointestinal endoscopy at the University Hospital of Malaysia (Kuala Lumpur) and the Changi General Hospital (Singapore) in the period between January 2004 and April 2007. Subjects who had been prescribed antibiotics, non-steroidal anti-inflammatory drugs (NSAIDs) or acid suppressants in the two-month period prior to recruitment as well as subjects known to be infected with the Human Immunodeficiency Virus (HIV) were excluded.

The case group comprised 12 patients newly diagnosed with primary non-cardia GC (International Classification of Diseases, 9^th^ revision, code 151). Subjects diagnosed with FD (n = 20) — defined as persistent or recurrent symptoms (pain or discomfort centred in the upper abdomen) in the absence of organic disease, in accordance with Rome II classification system, were invited to participate as controls. An additional four subjects presenting with gastric ulcers were recruited; however, their results were not reported on in the main text due to low numbers. Information pertaining to gender and age was obtained from each subject through a brief socio-demographic questionnaire.

### Sample collection

Antral gastric biopsies were obtained from symptomatic subjects undergoing routine endoscopic examination of gastrointestinal symptoms. These biological specimens were frozen in liquid nitrogen immediately after collection and transported to Australia on dry ice, where they were stored at −80 °C until further processing. In addition, peripheral whole blood was collected from all study subjects to determine *H. pylori* status.

### Microbiota sequencing and analysis

RNA was extracted from biopsies using the Isolate II RNA mini kit (Bioline; Sydney, NSW, Australia) and cDNA synthesized using the Tetro cDNA synthesis kit (Bioline) according to the manufacturer’s instructions.

The 16S rRNA gene was amplified using the KAPA HiFi HotStart ReadyMix (95 °C for 3 min, 25 cycles of 95 °C for 30 s, 55 °C for 30 s, 72 °C for 30 s, followed by a final step of 72 °C for 5 min) and the earth microbiome primers (515F-806R). Indices and Illumina sequencing adapters were attached using the Nextera XT Index Kit according the manufacturer’s instructions. Amplicon sequencing was performed with Illumina MiSeq 2 × 250 bp chemistry (n = 16192 ± 2237 total clean reads/sample derived from a total of 774,558 raw sequences; 24.7% sequences filtered out) at the Ramaciotti Centre for Genomics. All relevant positive (bacterial DNA) and negative (blank) PCR controls as well as extraction controls (extraction kit buffers) were included in the experimental procedures. Raw reads were analyzed using the MiSeq standard operating procedures within Mothur v1.37.3^12,13^. Differences across α-diversity and phylogenetic diversity (according to Faith) measures were examined using GraphPad Prism 6 employing both subsampled (n = 2000 reads) and non-subsampled read counts, and reported patterns were consistent across both datasets. To determine global differences in microbial composition, multivariate analyses such as non-metric multidimensional scaling (nMDS), permutational MANOVA (PERMANOVA), and PERMDISP were performed on relative abundances using Primer-E^14^. To identify specific microbial taxa that differed significantly across conditions, Linear Discriminant Analysis Effect Size (LEfSe)^15^ was performed using the Galaxy web application^16^. PICRUSt^17^ was used to predict bacterial metabolic contributions and both global and specific changes were examined using Primer-E and LEfSe, respectively. SparCC^18^ correlations were calculated in Mothur v1.39.1^13^ and plotted using Cytoscape v3.5.1^19^ in an attempt to identify bacterial networks within the gastric microbiota.

### *Helicobacter pylori* detection in serum

Detection of human IgG antibodies to *H. pylori* was performed using the MPD Helico Blot 2.1 kit (MP Biomedicals, Australia) according to the manufacturer’s instructions. In addition, this kit includes recombinant antigens with high predictive value for the indication of current *H. pylori* infection. This kit has been shown to exhibit higher sensitivity and specificity when compared to commercial ELISAs^20^.

### Data Availability

Raw data (fastq files) have been submitted to the European Nucleotide Archive (Accession number: PRJEB21497). Patient metadata and relative abundance are provided in Supplementary File S1.

## Results

### Impact of *Helicobacter pylori* infection and disease on α- and phylogenetic diversity of gastric microbiota

**Figure 1:**
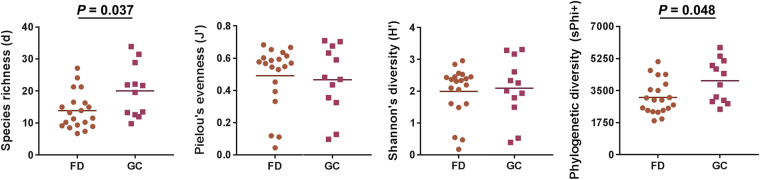
Alpha-diversity and phylogenetic diversity of the gastric microbiota. Phylogenetic diversity was calculated according to Faith. *P*-values were calculated using one-way ANOVA with a Tukey’s multiple comparisons test.

Species richness and phylogenetic diversity were found to be significantly increased in GC patients as compared to FD controls (Fig. 1). Evenness and Shannon’s diversity did not differ across subgroups (Fig. 1). *H. pylori* seropositive patients also had increased species richness and phylogenetic diversity but not evenness and Shannon’s diversity (Supplementary File 1: Figure S1A). Evidence that this was not due to an artefact arising from higher seropositivity levels in GC patients comes from the finding that FD controls positive for *H. pylori* had higher species richness and phylogenetic diversity as compared to FD controls that were seronegative (Supplementary File 1: Figure S1B). Further, these analyses indicated that the increased richness and phylogenetic diversity in GC patients was independent of *H. pylori* seropositivity (Supplementary File 1: Figure S1B).

### Associations between *Helicobacter pylori* infection and disease and the composition of gastric microbiota

**Figure 2:**
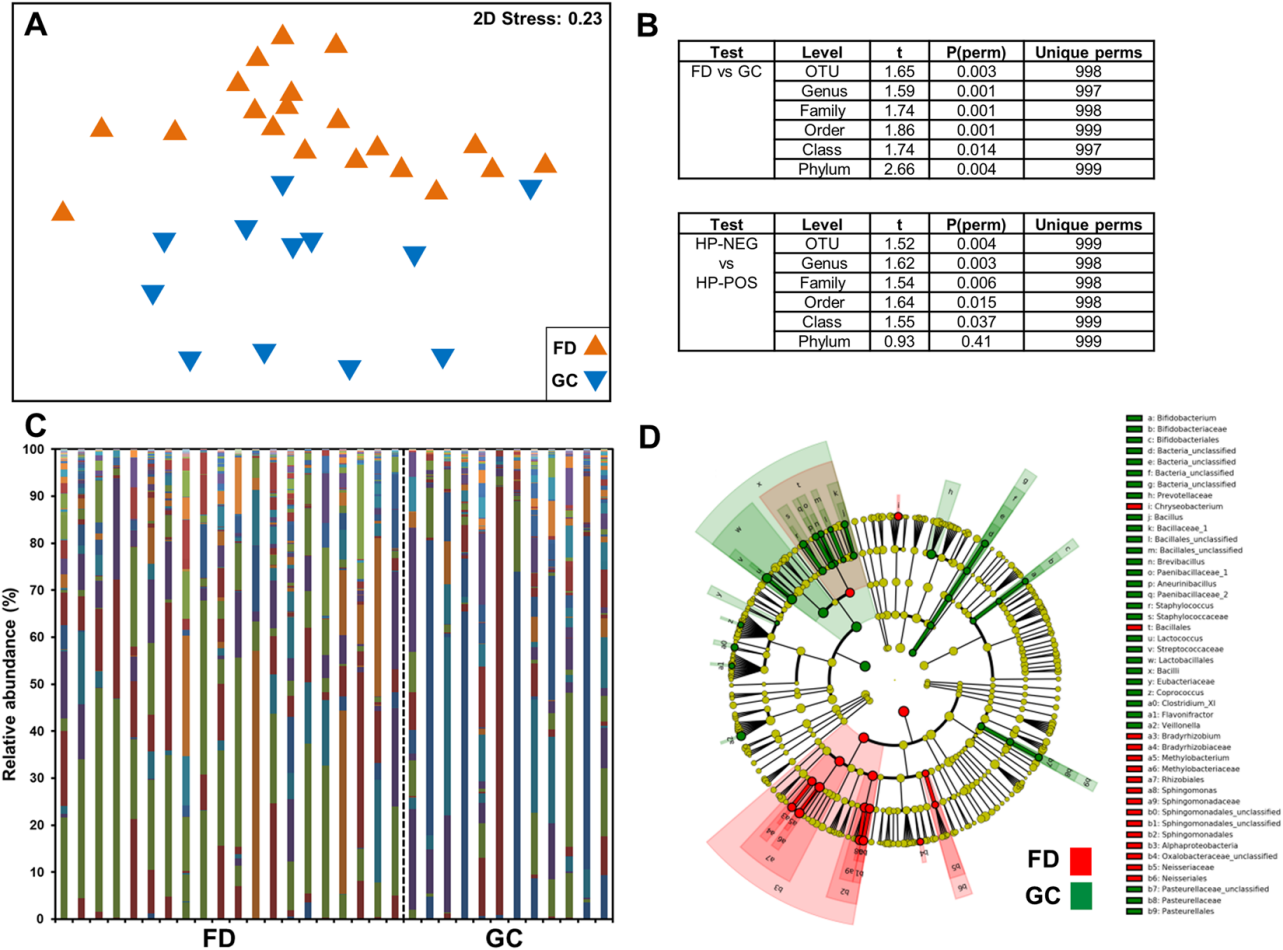
Composition of the gastric microbiota. (**A**) Non-metric multidimensional scaling plot and (**B**) PERMANOVA following square-root transformation and Bray-Curtis similarity resemblance. (**C**) Relative abundance of bacterial genera across FD and GC subjects following the removal of the contribution of *Helicobacter*. Colour legend is provided in Supplementary File 1. (**D**) Cladogram of LEfSe analysis between FD controls and GC patients nested for *H. pylori* serological status. Higher resolution colour legend is provided in Supplementary File 1. FD: Functional dyspepsia; GC: Gastric cancer; HP-NEG: negative for *H. pylori*; HP-POS: positive for *H. pylori*.

The global profile of the gastric microbiome was significantly altered during GC as compared to FD subjects (Fig. 2A–C), with differences observed up to the phylum level (Fig. 2B). *H. pylori* serological status of the subject also appeared to have a significant impact, with the gastric microbiota composition in subjects testing positive for the bacterium being different to those testing negative (Fig. 2B). Despite this, the differences observed between FD controls and GC patients, were independent of serological status, given that these comparisons showed significant differences when tested only in subjects that were *H. pylori* positive by serology (Supplementary File 1: Figure S2A). Further, the differences across disease and serological status did not arise from the relative abundance of *H. pylori* in the samples, as removal of the contribution of *Helicobacter* to the overall composition showed no differences in the results (Supplementary File 1: Figures S2B and S3).

We observed no significant differences in dispersion of the data (PERMDISP) across disease, serological status, indicating that the significant differences arising from the PERMANOVA analyses were locational and not dispersion effects. We also confirmed that the differences observed in our cohort did not arise due to age, gender or country of origin. PERMANOVA at both OTU and genus taxonomic levels comparing the gastric microbiome of FD patients from Singapore and Malaysia found no significant differences (OTU: t = 0.97, *P* = 0.48, Permutations = 992, Df = 18; Genus: t = 0.98, *P* = 0.45, Permutations = 993, Df = 18). No significant differences were found between male and female FD patients using PERMANOVA (OTU: t = 0.88, *P* = 0.76, Permutations = 995, Df = 18; Genus: t = 0.88, *P* = 0.72, Permutations = 995, Df = 18) and no correlation with age was observed using distance-based linear modelling (OTU: Pseudo-F = 1.32, *P* = 0.14, Df = 18; Genus: Pseudo-F = 1.28, *P* = 0.21, Df = 18).

**Figure 3:**
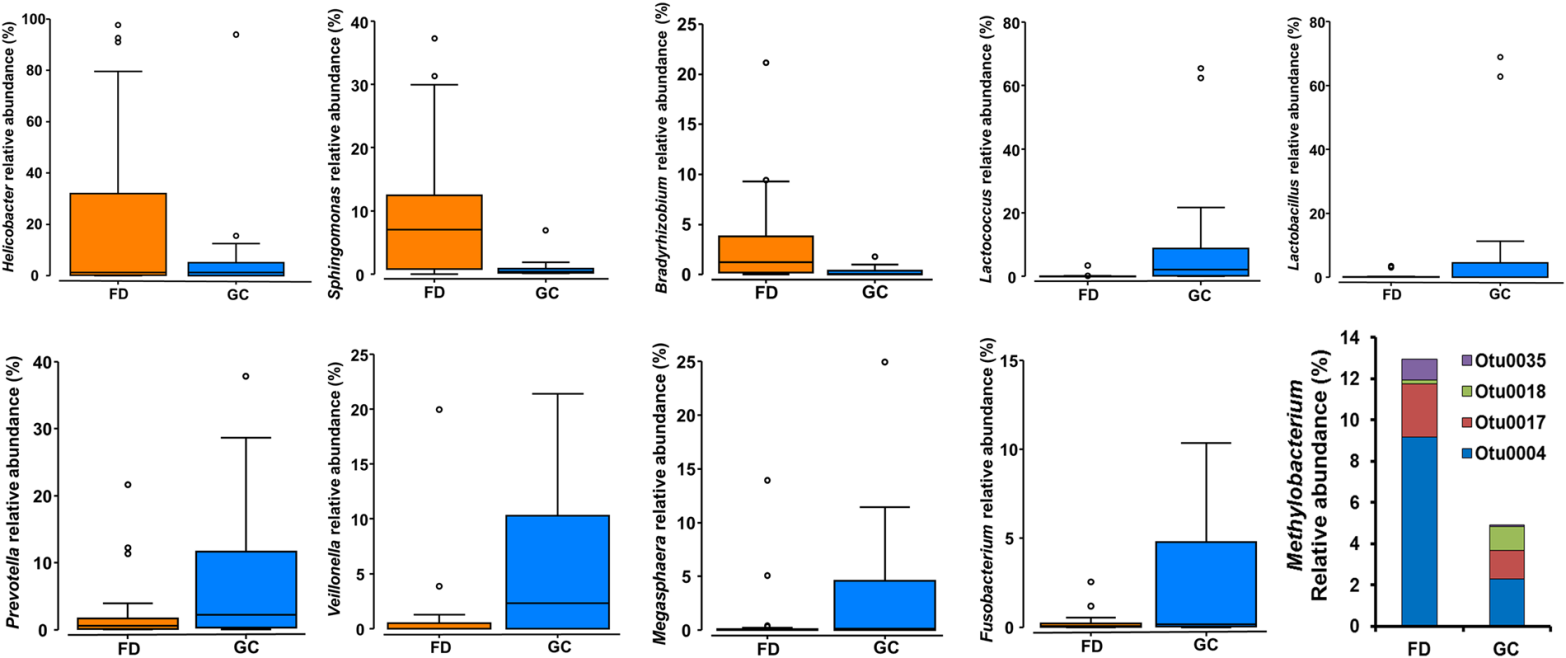
Relative abundance of bacterial genera of interest in FD controls and GC patients. *Methylobacterium* relative abundance was broken down to OTU contribution.

We then identified the bacterial taxa contributing to these differences using LEfSE (Fig. 2D, Supplementary File S1). 91 taxa were different when comparing FD controls and GC patients (Supplementary File S1: LEfSe2). *H. pylori* serological status was also found to enrich for a range of bacterial taxa, with 36 taxa enriched in negative subjects and 28 in positive subjects (Supplementary File S1: Figure S4, LEfSe6). Given this, we conducted a further comparison between FD controls and GC patients with serological status nested as a subclass. Thirty-eight bacterial taxa were identified to be significantly different between GC patients and FD controls (Fig. 2D, Supplementary File S1: LEfSe5), 23 of which were enriched in GC. These included *Lactococcus* OTU0002 (100% similarity to *Lactococcus lactis*), *Fusobacterium* OTU0087 (100% similarity to *Fusobacterium mortiferum*), *Pasteurellaceae_*unclassified OTU0022 (100% similarity to *Haemophilus parahaemolyticus* and *H. sputorum*), *Staphylococcus* OTU0045 (100% similarity to up to seven *Staphylococcus* species), and *Methylobacterium* OTU0018 (99% similarity to *Methylobacterium adhaesivum*). Other bacterial taxa of interest identified to be enriched in GC in the non-nested analysis (Supplementary File S1: LEfSe2) include *Veillonella* OTU0005 (100% similarity to *Veillonella atypica* and *V. dispar*), *Dialister* OTU0132 (100% similarity to *Dialister pneumosintes*), and *Leptotrichia* OTU0042 (100% similarity to *Leptotrichia buccalis*). The relative abundances of bacterial genera showing large changes across disease were plotted in Fig. 3. Most OTUs corresponding to *Methylobacterium* were found to be decreased in GC except for OTU0018 (Fig. 3).

### Associations between *Helicobacter pylori* infection and disease and the predicted bacterial metabolic contribution

Bacterial metabolic contributions were then predicted using PICRUSt and nearest sequence Taxon index (NSTI) calculated for these predictions (Supplementary File S1: PICRUSt counts, PICRUSt NSTI scores). The average NSTI score for all samples was 0.039 ± 0.004 which is well below the accepted threshold of 0.1, suggesting that the predictions were reliable.

**Figure 4:**
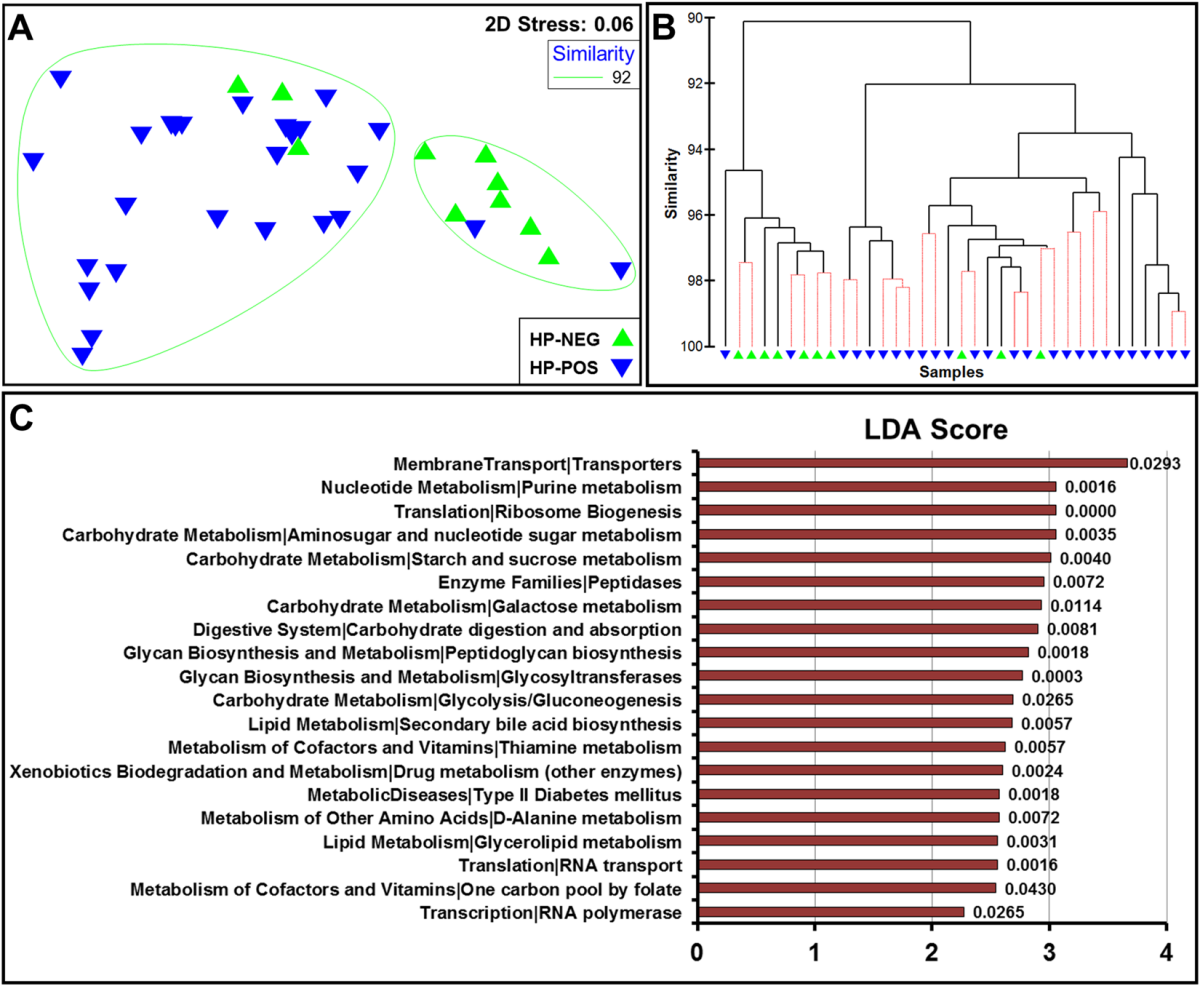
Prediction of the metabolic contribution of the gastric microbiota using PICRUSt. (**A**) Non-metric multidimensional scaling plot and (**B**) Cluster analysis with SIMPROF testing following Log(X + 1) transformation and Bray-Curtis similarity resemblance. (**C**) KEGG pathways (Level 3) enriched in GC using LEfSe analysis. *P*-values are presented at the end of the bars.

Serological status of the subject but not disease status was associated with a significantly shift in the predicted global microbial metabolic output. Subjects that were negative or positive were divided into two separate clusters (Fig. 4A,B). Despite some inconsistencies in the clusters, PERMANOVA identified significant differences between negative and positive subjects (t = 3.05, P(Perm) = 0.002, Permutations: 998). In contrast, no significant difference was found across disease (FD vs. GC: t = 1.48, P(Perm) = 0.12, Permutations: 999).

Predicted KEGG pathways enriched across disease and *H. pylori* serological status were then identified using LEfSe (Supplementary File S1: PICRUSt LEfSe1-5). Both simple and nested designs were performed to account for confounding factors. We observed 20 predicted pathways (KEGG Level 3) to be enriched in GC patients when compared to FD controls (serological status nested as subclass, Fig. 4C). A complete list of enriched pathways is provided in Supplementary File S1 (PICRUSt LEfSe3). Of interest, a number of predicted pathways related to bacterial carbohydrate metabolism were found to be enriched in GC (Fig. 4C). Moreover, carbohydrate digestion and absorption, which is partly responsible for the production of short chain fatty acids (SCFAs) such as butyrate, propionate and acetate, was also enriched in GC (Fig. 4C).

### Identification of bacterial interactions in gastric carcinogenesis

**Figure 5:**
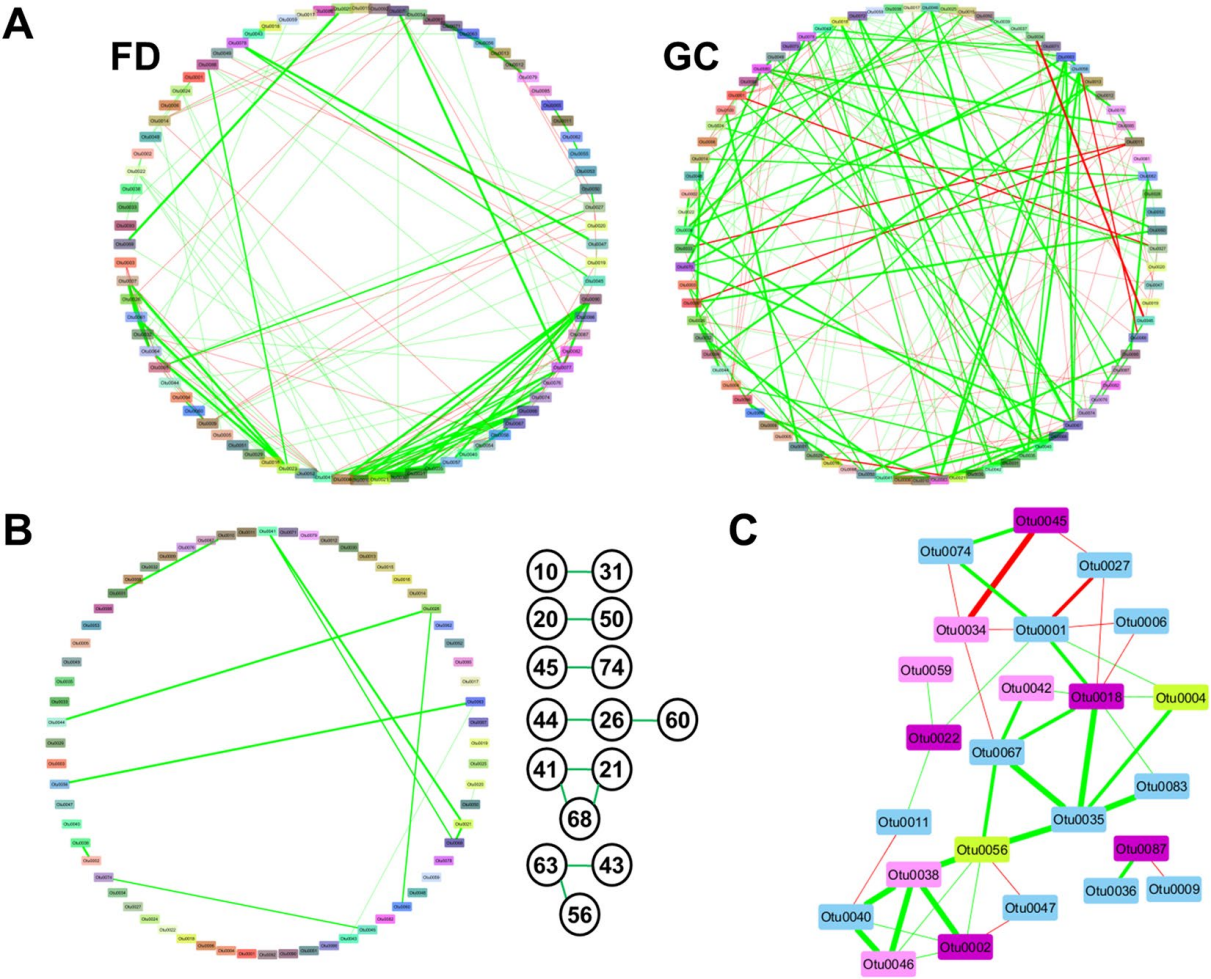
Network analysis of bacterial OTUs within the gastric microbiota. (**A**) Network plots for FD controls and GC patients. Correlations for OTUs 1–100 were calculated using SparCC and correlations greater than 0.6 or lower than −0.5 were visualized using Cytoscape. Co-occurrence and co-exclusion interactions were drawn in green and red, respectively. Thickness of the line indicates strength of the correlation. Full lists of correlations are provided in Supplementary File 1. (**B**) Similarities between the FD and GC networks, and the shared interactions between OTUs. Numbers represent OTUs that interact with each other (i.e. 10 = OTU0010). (**C**) Network analysis of OTUs that were identified to be enriched in GC using LEfSe. OTUs in dark purple were identified as enriched in GC in the nested analysis; OTUs in pink were identified as enriched in GC in the non-nested analysis; OTUs in yellow were enriched in FD in either the nested or non-nested analysis; OTUs in light blue were not found to be enriched using LEfSe. A full list of correlations is provided in Supplementary File 1.

To determine the interactions across different bacterial OTUs, we then utilised SparCC and Cytoscape to calculate and visualize correlations among the first 100 OTUs. Interaction network across bacterial OTUs was found to be notably denser in GC patients as compared to FD controls (Fig. 5A, Supplementary File S1: SparCC FD, SparCC GC). This could be related to an increase in abundance of other organisms following the decreased dominance of *H. pylori* in GC patients (Fig. 3). Comparison of the FD and GC networks to identify shared interactions (independent of correlation direction) between OTUs found six interactions across 15 OTUs to be common across the networks (Fig. 5B). The relationships between OTU0011 (*Lactobacillus*) and OTU0031 (*Clostridium* sensu stricto), OTU0021 (*Prevotella*), OTU0041 (*Faecalibacterium*), and OTU0068 (*Megamonas*), OTU0021, OTU0041, and OTU0068, as well as OTU0043, OTU0056 and OTU0063 were all consistent across FD and GC networks. In contrast, the correlations between OTU0020 and OTU0050, and OTU0045 and OTU0074 were in opposite directions in the FD and GC networks.

To define the network most relevant to disease, we then built a network comprising the five OTUs (OTU0002, OTU0018, OTU0022, OTU0045, and OTU0087) that were found to be enriched in GC patients in the nested analysis (Supplementary File S1: LEfSe5), included their interaction partners, and if their interaction partners had been associated with disease in the non-nested analysis (Supplementary File S1: LEfSe2). We visualized the network in Cytoscape (Fig. 5C) and the list of correlations is provided in the supplementary material (Supplementary File S1: SparCC GC LEfSe). One notable interaction node in this GC network is that among OTU0002 (*Lactococcus*), OTU0038 (*Aneurinibacillus*) and OTU0046 (*Bacillus*). Further, the co-exclusion interaction between OTU0001 (*Helicobacter*) and OTU0027 (100% similarity to *Campylobacter concisus*) is likely to be relevant in the context of gastrointestinal disease.

## Discussion

It is widely accepted that GC is a multifactorial disease involving the interplay of host, microbial and environmental factors. Despite *H. pylori* being recognised as the main etiological factor in the development of GC, and the ability to eradicate this organism using triple or quadruple regimens, GC remains the third leading cause of cancer-related deaths worldwide. With this in mind, studies have focussed on host genetics, and more recently, the gastric microbiome’s contributions to disease etiology. Here, the viable gastric microbiome was profiled in FD and GC subjects to identify microbial changes across disease that may be relevant to gastric carcinogenesis.

Previous studies have reported conflicting results on the α-diversity of the gastric microbiome across the GC cascade^6,7,9^. To shed light on these discrepancies, we examined all the different α-diversity measures in detail and found species richness but not evenness or Shannon’s diversity to increase in GC. Phylogenetic diversity was found to follow similar patterns as those observed for species richness. It is likely that large fluctuations in *H. pylori* relative abundances across subjects contribute to highly variable evenness measures but do not impact richness measures. In support of this conclusion, a strong inverse association between the relative abundance of *H. pylori* and α-diversity has been previously reported^8^. We did identify serological status as an important contributor, with *H. pylori*-positive subjects exhibiting higher species richness and phylogenetic diversity. These results are of significance as they suggest that the ability of the infection to illicit an immune response in the host plays an important role in shaping the composition of the gastric microbiome.

The global composition of the gastric microbiome in our cohort was also significantly different in GC as has been previously observed^6,8–10^; however, we were able to confirm that these differences were independent of *H. pylori* contribution to the overall relative abundance. However, serological status was associated with a significant shift in the gastric microbiome composition. Importantly, the differences across gastric carcinogenesis and *H. pylori* serological status were independent of each other, highlighting the importance of host- *H. pylori* interactions, but also suggesting a role for the gastric microbiome in disease progression. Notably, there was an increase in co-occurrence interactions across the gastric microbiome in GC as compared to FD. While this may arise from the depletion of *H. pylori* in GC allowing other microbial species to flourish, it may also lead to other consequences in the host, with one recent study reporting an increased bacterial load in GC patients as compared to chronic gastritis patients^9^.

A marked increase in the relative abundance of lactic acid producing bacteria (*Lactococcus* and *Lactobacillus*) was observed in GC patients. Further, *Lactococcus* OTU0002 was found to have strong co-occurrence interactions with two other OTUs associated with GC (*Aneurinibacillus* OTU0038 and *Bacillus* OTU0046). Indeed, previous studies have observed a similar increase in abundance of *Lactobacillus* species in GC^6,7,9^. While *Lactobacillus* species are often utilized as probiotics and assumed to be beneficial to the host, in the context of cancer, elevated levels of lactic acid can be highly detrimental. For example, lactate can serve as an energy source for tumor cells, inducing glycolytic enzymes which leads to an increase in ATP supply; this metabolite can also promote inflammation and stimulate tumor angiogenesis^21–24^. Of particular significance, Lertpiriyapong *et al*.^5^, using a transgenic *H. pylori* INS-GAS mouse model, showed that colonisation with microbiota limited to three species of Altered Schaedler’s Flora, including ASF356 (*Clostridium* species), ASF361 (*Lactobacillus murinus*) and ASF519 (*Bacteroides* species), were sufficient to induce neoplasia. Most studies have focussed on the role of host lactic acid production in cancer cell metabolism, but these findings suggest that at least in relation to GC, the role of exogenous bacterial lactic acid should be investigated further.

An increased abundance of *Escherichia*/*Shigella* was observed in our GC patients, consistent with previous findings^9^. It is difficult to conclude what role this may play in GC given the diversity in metabolic and pathogenic potential within this group of organisms. However, specific genotoxin strains or invasive pathotypes of *E. coli* have been linked with inflammatory bowel diseases (IBD)^25^ and colorectal cancer^26^. Thus, the increased abundance of *Escherichia*/*Shigella* has the potential to be harmful to the host.

Bacterial species commonly found in the oral cavity including *Fusobacterium*, *Veillonella*, *Leptotrichia*, *Haemophilus*, and *Campylobacter* were found to have higher relative abundances in GC patients. This enrichment of oral organisms is increasingly being reported across several types of cancer such as esophageal adenocarcinomas, colorectal adenocarcinomas, and breast cancers^27–29^. Of these, *Fusobacterium* species have received a lot of attention due to their pro-inflammatory nature, with TLR4 and autophagy playing a very important role in the inflammation they induce^30–32^. Notably, polymorphisms in TLR4 and autophagy are well known to increase the risk of developing GC in Chinese individuals^33–36^, thus, it would be interesting to determine the impact of these polymorphisms in the context of increased levels of *Fusobacterium*. Interestingly, a recent analysis of the gastric microbiome in two populations from Colombia (Túquerres and Tumaco), found *Leptotrichia* and *Veillonella* to be significantly more abundant in inhabitants of Túquerres, these individuals exhibiting a 25-fold higher risk of GC than Tumaco inhabitants despite both populations having similar *H. pylori* prevalence^11^. *Fusobacterium* species have been shown to co-aggregate with other bacteria to form biofilms that play a key role in colorectal adenocarcinoma initiation and progression^29^. In this context, *Veillonella* species could be a plausible pro-oncogenic partner as these bacteria have been found to be increased in oral, lung and colorectal cancer patients^37–39^. Given this, the enrichment of these oral bacterial species should not only be further investigated in GC, but also in other types of cancer.

An additional interaction of interest was the co-exclusion relationship between *Helicobacter* OTU0001 and *Campylobacter* OTU0027 (putatively identified as *C. concisus*). Emerging *Campylobacter* species such as *C. concisus* are members of the oral microbiota that have been associated, through epidemiological studies, with a range of gastrointestinal diseases^28,40,41^. A recent meta-analysis on the relationship between *H. pylori* and IBD hypothesized that a co-exclusion relationship between this gastric bacterium and related members of the *Helicobacter* and *Campylobacter* genera in the intestinal tract may be one mechanism by which *H. pylori* exerts a protective effect in IBD^42^.

Another important finding was the effect of *H. pylori* serological status on the predicted global metabolic contribution of the gastric microbiome, an effect that was not observed in disease. This highlighted the potential capacity for the immune response against *H. pylori* to regulate the overall composition and predicted metabolic output of the gastric microbiome. We did observe a number of bacterial metabolic pathways that were predicted to be significantly enriched in GC. In addition to an array of carbohydrate metabolism pathways likely related to the enrichment of *Lactococcus* and *Lactobacillus* species in GC, we identified enrichment of carbohydrate digestion and absorption, which is involved in the production of SCFAs such as butyrate, propionate and acetate. Importantly, increased levels of bacterial SCFAs have been shown to induce hyperproliferation of colonic cells^43^, and have been proposed to induce oesophageal trans-differentiation in Barrett’s esophagus^44^. However, the results derived from PICRUSt are predictive in nature and should be interpreted with caution.

This study is not without limitations. Despite the significant differences identified, this is a pilot study with a low number of subjects. Additional studies with a larger cohort of subjects are warranted. Further, it remains to be seen whether the changes in the gastric microbiome are involved in the progression of disease or are a consequence of disease.

In conclusion, the viable gastric microbiome appears to be significantly affected by *H. pylori* serological status and altered in gastric carcinogenesis. Our study identifies a number of potential mechanisms of interest such as enrichment of pro-inflammatory oral bacterial species, increased abundance of lactic acid producing bacteria, and enrichment of SCFA production pathways. Further investigations should focus on whether these changes are a cause or consequence of GC.

## Electronic supplementary material

Supplementary file 1
